# Preparation of a Porous Composite Film for the Fabrication of a Hydrogen Peroxide Sensor

**DOI:** 10.3390/s110605873

**Published:** 2011-05-31

**Authors:** Mu-Yi Hua, Chun-Jen Chen, Hsiao-Chien Chen, Rung-Ywan Tsai, Wen Cheng, Chun-Lin Cheng, Yin-Chih Liu

**Affiliations:** 1 Green Technology Research Center, Department of Chemical and Materials Engineering, Chang Gung University, Tao-Yuan 33302, Taiwan; E-Mails: d9023006@msn.com (C.-J.C.); chainjim@yahoo.com.tw (H.-C.C.); jordan9090@mail2000.com.tw (Y.-C.L.); 2 Biosensor Group, Biomedical Engineering Research Center, Chang Gung University, Tao-Yuan 33302, Taiwan; 3 Electronics and Optoelectronics Research Laboratories, Industrial Technology Research Institute, Hsinchu 31040, Taiwan; E-Mail: ry_tsai@itri.org.tw; 4 Department of Chemical Engineering, Chung Yuan University, Tao-Yuan 33023, Taiwan; E-Mails: b750214@hotmail.com (W.C.); clcheng1688@gmail.com (C.-L.C.)

**Keywords:** polyaniline, polyacrylic acid, enzyme-free, hydrogen peroxide sensor

## Abstract

A series of dopant-type polyaniline-polyacrylic acid composite (PAn-PAA) films with porous structures were prepared and developed for an enzyme-free hydrogen peroxide (H_2_O_2_) sensor. The composite films were highly electroactive in a neutral environment as compared to polyaniline (PAn). In addition, the carboxyl group of the PAA was found to react with H_2_O_2_ to form peroxy acid groups, and the peroxy acid could further oxidize the imine structure of PAn to form *N*-oxides. The *N*-oxides reverted to their original form via electrochemical reduction and increased the reduction current. Based on this result, PAn-PAA was used to modify a gold electrode (PAn-PAA/Au) as a working electrode for the non-enzymatic detection of H_2_O_2_. The characteristics of the proposed sensors could be tuned by the PAA/PAn molar ratio. Blending PAA with PAn enhanced the surface area, electrocatalytic activity, and conductivity of these sensors. Under optimal conditions, the linear concentration range of the H_2_O_2_ sensor was 0.04 to 12 mM with a sensitivity of 417.5 μA/mM-cm^2^. This enzyme-free H_2_O_2_ sensor also exhibited a rapid response time, excellent stability, and high selectivity.

## 1. Introduction

Hydrogen peroxide (H_2_O_2_) is a chemical used widely in the food, pharmaceutical, paper, and chemical industries. H_2_O_2_ is also one of the products of reactions catalyzed by oxidase enzymes in many biological and environmental processes [1]. However, it is also a reactive oxygen species that can cause functional and morphological disturbances as well as cancer when produced in excess in the human body [2,3]. Therefore, the development of a rapid, accurate, and reliable sensor for the detection of H_2_O_2_ is important. Traditional techniques, such as titrimetry, fluorescence, and absorbance spectroscopy, have been explored [4–8]. Recently, a new generation of sensors based on electrochemical technology has been extensively employed for the design of H_2_O_2_ sensors due to their simplicity, high sensitivity, and selectivity [9,10]. Particularly, enzyme-based electrochemical sensors, such as horseradish peroxidase (HRP), hemoglobin (Hb), and myoglobin (Mb), have attracted increasing attention due to their intrinsic selectivity. However, their performance strongly depends on the activity of enzymes that are relatively expensive and unstable [11–13]. Consequently, the most recent studies have sought to directly determine H_2_O_2_ using enzyme-free electrodes [14–20].

The conducting polymer polyaniline (PAn) has attracted significant attention to the development of sensors due to its good biocompatibility and high electrical conductivity [21,22]. Various enzymes, including glucose oxidase [23], HRP [24], and cholesterol oxidase [25], have been immobilized on PAn using electrochemical polymerization or covalent binding [26]. PAn is an effective mediator for electron transfer in redox or enzymatic reactions and can also be used as a suitable matrix for the immobilization of biomolecules [27]. However, PAn exhibits electroactivity only in an acidic medium (pH < 4), which significantly limits its potential applications [28]. To overcome this limitation, many types of acid-doped PAn compounds have been synthesized that maintain their electroactivity in a neutral medium [29–31].

Polyacrylic acid (PAA) is a good dopant candidate due to the presence of the carboxyl group on the side chain. Dopant-type PAn can be prepared directly by blending PAn with PAA to form a PAn-PAA composite without any chemical modification. These composites have been used in electrochemical capacitors, separation applications, and sensors [32–34]. However, no studies have been reported for PAn-PAA-modified electrodes that can be used to detect H_2_O_2_ non-enzymatically in a neutral medium at lower potentials.

In this study, three types of PAn-PAA composites with different molar ratios were prepared and investigated. PAn was chemically oxidized by H_2_O_2_ in the presence of PAA to form *N*-oxide and was then returned to its native state by electrochemical reduction, which resulted in an increase of the reduction current. Thus, an enzyme-free H_2_O_2_ sensor based on a PAn-PAA-modified Au (PAn-PAA/Au) electrode was developed to detect H_2_O_2_ directly by assessing its reduction current. These sensors showed excellent sensitivity, a rapid response time, and high selectivity.

## 2. Experimental Section

### 2.1. Apparatus and Reagents

Aniline, hydrogen chloride, and ammonium persulfate were purchased from Merck; chloroform was purchased from Fisher Sci., H_2_O_2_ (30%), and PAA (MW: 280,000 Da) were purchased from Showa, and dimethyl sulfoxide (DMSO) was purchased from Tedia. The supporting electrolyte consisted of 0.2 M phosphate buffer solution (PBS) at pH 7.0. All aqueous solutions were prepared in deionized (DI) water.

Fourier transform infrared (FT-IR) spectra were obtained using a Bruker-Tensor 27 spectrometer at a spectral resolution of 8 cm^−1^. X-ray photoelectron spectroscopy (XPS) measurements were performed using a VG Scientific ESCALAB 250 system. Absorption spectra were obtained using an ultraviolet-visible near IR (UV-Vis-NIR) spectrometer (Perkin Elmer, Lambda 800/900). Field-emission scanning electron microscopy (FE-SEM) was performed using a Hitachi S-5000 system. Electrochemical measurements were performed on a CHI 660 A electrochemical workstation (CH Instruments, USA) with a three-electrode system consisting of the PAn-PAA/Au electrode, a bare Au electrode and an Ag/AgCl electrode as the working, counter and reference electrodes, respectively. All electrochemical measurements were performed in 40 mL of 0.2 M PBS at pH 7.0 and 25 °C.

### 2.2. Procedures

#### 2.2.1. Preparation of the PAn-PAA Composites and Their Oxidation by H_2_O_2_

The chemical polymerization of PAn was performed according to a previous report [35,36]. PAn (10 mmol) was dissolved in 50 mL of DMSO. Aliquots of 2 mL, 3 mL, and 5 mL of this solution were added to 8 mL, 7 mL, and 5 mL of PAA solution, respectively, which contained 10 mmol of PAA per 50 mL of DMSO. After stirring for 2 h, colloidal suspensions of PAn-PAA28, PAn-PAA37, and PAn-PAA55 with PAn/PAA molar ratios of 2/8, 3/7, and 5/5, respectively, were obtained. H_2_O_2_ (0.5 mmol) was added to 1 mL of these colloidal suspensions, and the solutions were incubated in a 43-kHz ultrasonic bath at 40 °C for 0.5 h in a dark room. After purification and vacuum-drying at 35 °C for 24 h, the oxides of the composites were obtained.

#### 2.2.2. Preparation of the PAn-PAA/Au Electrodes

To prepare the PAn-PAA/Au electrodes, 5 μL of each colloidal suspension was dropped onto an Au disk electrode (0.1 cm^2^) and dried in a vacuum aspirator at 30 °C for 24 h. The electrodes were washed with DI water before use.

## 3. Results and Discussion

### 3.1. Characterization of the PAn-PAA Composite Films

**Figure 1.**
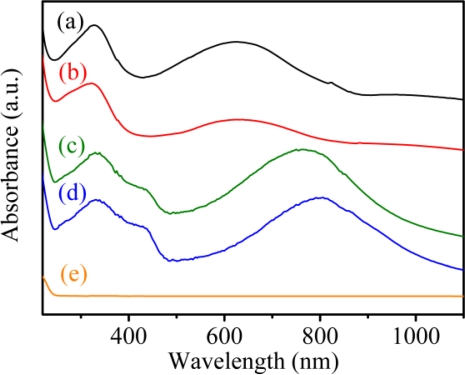
UV-Vis-NIR absorption spectra of **(a)** PAn, **(b)** PAn-PAA55, **(c)** PAn-PAA37, **(d)** PAn-PAA28 and **(e)** PAA.

The UV-Vis-NIR absorption spectrum of solid PAn exhibited two absorption peaks at 328 nm and 625 nm (Figure 1(a)), which originated from the π–π* transitions within the benzene ring and quinoid exciton bands, respectively [37]. In the PAn-PAA composite, the intensity of the peak associated with the quinoid ring of PAn decreased as the molar fraction of PAA increased (Figure 1(b–d)). When the molar fraction of PAn/PAA was 3/7, the peak intensity of the quinoid ring nearly disappeared, which was due to the protonation of the imine groups. Meanwhile, the quinoid ring next to the protonated imine became a semiquinoid radical cation, which decreased the intensity of the exciton absorption peak and generated the polaron/bipolaron absorption peaks at 437 and 762 nm [38]. The doping level of PAn increased as the PAA concentration increased, which is reflected in the increasing intensity of the polaron and bipolaron absorption peaks.

**Figure 2.**
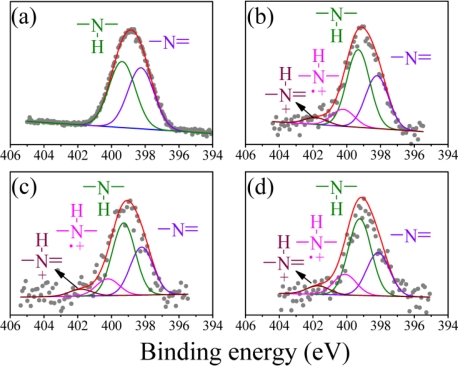
N (1s) XPS spectra of **(a)** PAn, **(b)** PAn-PAA55, **(c)** PAn-PAA37 and **(d)** PAn-PAA28.

The N (1s) XPS spectrum of the undoped PAn can be deconvoluted into two peaks: the imine peak at 398.2 eV and the amine peak at 399.3 eV (Figure 2(a)). The area ratio of the two peaks was close to 1, which is consistent with the theoretical value reported in the literature [39]. In addition to the two peaks, two positively charged nitrogen peaks from the PAn-PAA composites were generated at 400.2 eV for $–\overset{\overset{H}{|}}{\underset{•+}{N}}–$ and 401.8 eV for $–\overset{\overset{H}{|}}{\underset{+}{N}}=$, which were identified as the polaron and bipolaron, respectively (Figure 2(b–d)) [39]. Moreover, the polaron and bipolaron peak areas increased as the PAA concentration increased, indicating that the doping level of PAn increased as the amount of added PAA increased. These results further demonstrated the successful preparation of the doping-type of PAn-PAA composites and are consistent with the UV-Vis-NIR absorption spectra shown in Figure 1.

### 3.2. Surface Morphologies of the PAn-PAA/Au Electrodes

**Figure 3.**
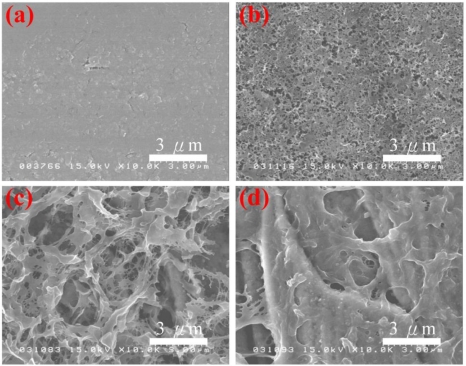
FE-SEM micrographs of **(a)** PAn/Au, **(b)** PAn-PAA55/Au, **(c)** PAn-PAA37/Au and **(d)** PAn-PAA28/Au electrodes.

The surface morphologies of the PAn/Au and PAn-PAA/Au electrodes are shown in Figure 3. The PAn/Au electrodes displayed a smooth surface. However, the pore size of the PAn-PAA/Au electrodes increased as the PAA content increased. It is well known that PAA is a water-soluble polymer that can be washed off from the composites when it is in excess (*i.e.*, when it does not blend well or does not interact with PAn). At lower ratios of PAA/PAn, most of the PAA interacted with PAn, which resulted in a lower amount of PAA removed by washing, and the electrode surface exhibited smaller, dense pores (Figure 3(b)). In contrast, at higher ratios of PAA/PAn, most of the PAA did not blend with the PAn and was washed off, forming a smaller amount of larger pores.

### 3.3. Electrochemical Behavior of the PAn-PAA/Au Electrodes

**Figure 4.**
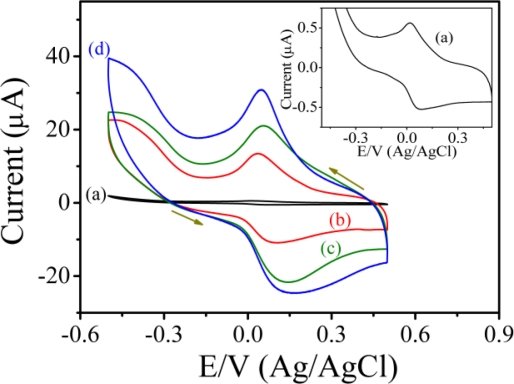
Cyclic voltammograms of **(a)** PAn/Au, **(b)** PAn-PAA55/Au, **(c)** PAn-PAA37/Au and **(d)** PAn-PAA28/Au electrodes.

**Figure 5.**
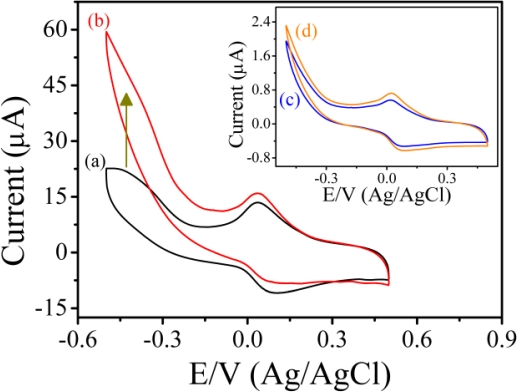
Cyclic voltammograms of the PAn-PAA55/Au electrode in the **(a)** absence and **(b)** presence of 2 mM H_2_O_2_. Inset: Cyclic voltammograms of the PAn/Au electrode in the **(c)** absence and **(d)** presence of 2 mM H_2_O_2_.

Figure 4 shows the typical cyclic voltammograms of PAn/Au and PAn-PAA/Au electrodes in PBS at pH 7.0 at a scan rate of 0.05 V/s. For the PAn/Au electrode, a pair of well-defined redox peaks were observed at 0.077 and 0.018 V (Ag/AgCl), which were attributed to the electrochemistry of the leucoemeraldine/leucoemeraldine radical cations (Figure 4, inset) [40]. However, when compared to the PAn-PAA/Au electrodes, the redox peaks of PAn were much smaller and difficult to detect, indicating that the PAn showed poor electroactivity in a neutral environment. In contrast, the PAn-PAA/Au electrodes exhibited considerable electroactive behavior due to the presence of the dopant-type PAn-PAA composites. The current of the redox peaks for the PAn-PAA/Au electrodes increased as the PAA content increased, because this increased the dopant levels of the PAn. The increase of PAn would increase the concentrations of PAn that could be doped by PAA. However, it also shows an association with the concentration of PAA. Although the composite of PAn-PAA55 may provide the highest PAn concentration, the lowest PAA level resulted in the lowest dopant capacity. In contrast, a higher dopant level is expected at higher PAA concentrations. These results were also in accordance with the findings from XPS analysis. In addition, the reduction current of the PAn-PAA55/Au electrode increased markedly from 0 V to −0.5 V (Ag/AgCl) with a concomitant decrease in the oxidation current in the presence of 2 mM H_2_O_2_ (Figure 5); no obvious change in the response current for the PAn/Au electrode was observed under the same conditions (Figure 5, inset).

**Figure 6.**
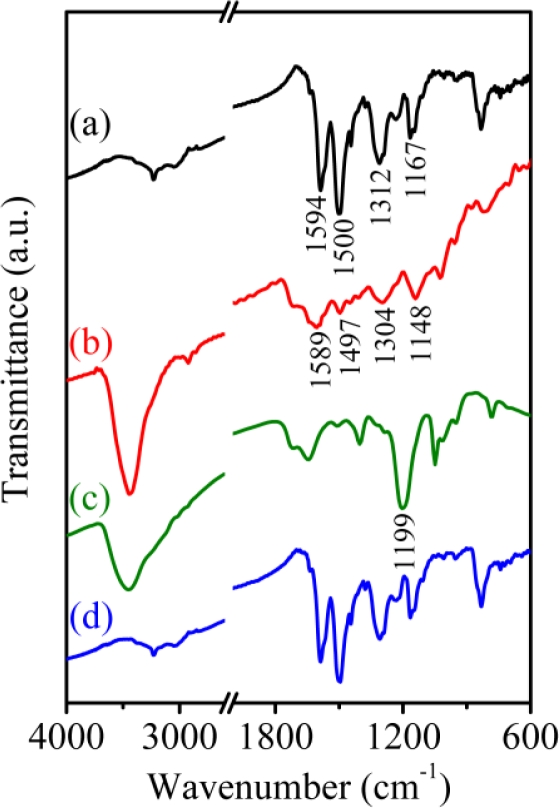
FT-IR spectra of **(a)** PAn, **(b)** PAn-PAA28, **(c)** PAn-PAA28 and **(d)** PAn after reacting with 2 mM H_2_O_2_.

The electrochemical behavior of the PAn/Au and PAn-PAA/Au electrodes in the absence and presence of H_2_O_2_ could be explained by their structures as measured by FT-IR (Figure 6). The characteristic peaks of PAn include the stretching vibrations (ν) of N=Q=N (ν_n=q=n_) at 1,594 cm^−1^, ν_n-b-n_ at 1,500 cm^−1^, ν_c-n_ at 1,312 cm^−1^, and a mode of N=Q=N at 1,167 cm^−1^ (Figure 6(a)) [37,41]. When blended with PAA, e.g., PAn-PAA28, all of the PAn characteristic peaks shifted to lower wavenumbers, indicating that delocalization likely occurred on the main chain of PAn after doping with PAA (Figure 6(b)). After reaction with H_2_O_2_ (Figure 6(c)), one strong additional peak caused by the resonance structure of *N*-oxide appeared at 1,199 cm^−1^; this structure may have been generated by the reaction of the carboxyl group of PAA with H_2_O_2_, which could form a peroxy acid group that would then oxidize the imine structure of PAn [42]. The *N*-oxide peak was difficult to observe for PAn in the presence of H_2_O_2_ because no peroxy acid was formed that could oxidize the imine to the *N*-oxide (Figure 6(d)). This result is consistent with previous results showing the formation of pyridine *N*-oxide by the addition of pyridine to a mixture of acetic acid and H_2_O_2_ [43]. The formation of *N*-oxide on the PAn-PAA/Au electrode caused an increase in the reduction current and a decrease in the oxidation current during the electrochemical process, as shown in Figure 5. Accordingly, a three-step mechanism can be proposed for the enzyme-free H_2_O_2_ sensors based on the PAn-PAA/Au electrodes, as shown below:

(step 1) $$\text{PAA}+{\text{H}}_{2}{\text{O}}_{2}\to \text{peroxy acid}$$

(step 2) PAn+peroxy acid→PAnN-oxide

(step 3) $$\text{PAn}N\text{-oxide}+{2\text{e}}^{-}+{2\text{H}}^{+}\to \text{PAn}+{\text{H}}_{2}\text{O}$$

First, added H_2_O_2_ chemically oxidizes the carboxyl group of PAA to form peroxy acid. Then the imine of PAn is chemically oxidized by the peroxy acid to form the imine *N*-oxide. Finally, each *N*-oxide reverts to an imine by accepting electrons and protons during the electrochemical reduction. This phenomenon yields the potential benefit of acting as a non-enzymatic H_2_O_2_ sensor.

### 3.4. Amperometric Determination of H_2_O_2_ Using the PAn-PAA/Au Electrodes

**Figure 7.**
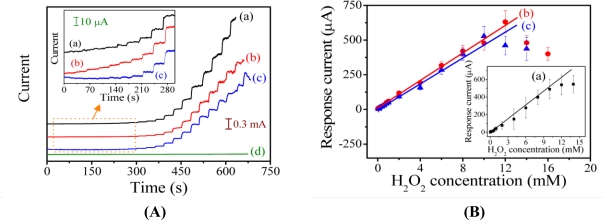
**(A)** Typical current-time responses of the **(a)** PAn-PAA28/Au, **(b)** PAn-PAA37/Au, **(c)** PAn-PAA55/Au and **(d)** PAn/Au electrodes with the addition of different concentrations of H_2_O_2_ in 0.2 M PBS at pH 7.0. (H_2_O_2_ concentrations were from 0.01, 0.02, 0.04, 0.06, 0.08, 0.1, 0.2, 0.4, 0.6, 0.8, 1, 2, 4, 6, 8, 10, 12, 14 and 16 mM, respectively). **(B)** Linear dependence of the response currents *vs.* the H_2_O_2_ concentrations for **(a)** PAn-PAA28/Au, **(b)** PAn-PAA37/Au and **(c)** PAn-PAA55/Au electrodes.

Figure 7(A) shows the amperometric response curves of the PAn-PAA/Au electrodes in the presence of H_2_O_2_ at an applied potential of −0.5 V (Ag/AgCl). With the successive addition of H_2_O_2_ in PBS (pH 7.0) at a stirrer rotation speed of 400 rpm, the response currents increased as the H_2_O_2_ concentration increased and achieved 95% of the steady-state current within 4.98 s, 4.76 s, and 3.18 s for the PAn-PAA28/Au, PAn-PAA37/Au and PAn-PAA55/Au electrodes, respectively. The corresponding calibration plots for the three amperometric response curves and the sensor performances are presented in Figure 7(B) and Table 1, respectively.

The current response to H_2_O_2_ of the PAn-PAA28/Au electrodes was linear over the range of 0.8 to 12 mM (R^2^ = 0.994) with a sensitivity of 553.9 μA/mM-cm^2^ (y = 55.39x) for eight measurements (n = 8). The detection limit was 0.04 mM at a signal-to-noise ratio of 3. The sensitivities of the PAn-PAA/Au electrodes increased as the PAA content increased, which is due to the porous structure of the composite films (Figure 3). The larger pore sizes and the increased number of pores offered more active surfaces for reaction with H_2_O_2_ and increased the electrode sensitivity. Furthermore, the higher electroactivity of PAn was enhanced as the PAA content increased. However, the highest amount of PAA may have caused swelling of the PAn-PAA28 composite after long-term application, and this behavior resulted in an unstable sensor that exhibited a large deviation in electrode response. After evaluating the performance of all of the electrodes, the sensor based on the PAn-PAA37/Au electrode was considered to be optimal with a sensitivity of 417.5 μA/mM-cm^2^ (y = 41.75x) and a linear range of 0.04 to 12 mM. Besides, the three electrodes also showed the repeatable property with the RSD values of 4.4% (PAn-PAA28/Au electrode), 3.5% (PAn-PAA37/Au electrode), and 3.8% (PAn-PAA55/Au electrode) at 1 mM of H_2_O_2_ concentration. Comparing with other literatures based on the similar detection mechanism [44,45], their sensitivities (417.5 μA/mM-cm^2^) were higher than those of PBBI/Au electrode (35.1 μA/mM-cm^2^) and PBI-BA/Au electrode (74.2 μA/mM-cm^2^), indicating that PAn-PAA modified Au electrodes were more sensitive to H_2_O_2_.

### 3.5. Selectivity and Stability of the PAn-PAA37/Au Electrodes

**Figure 8.**
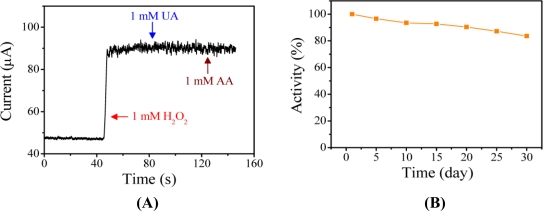
**(A)** Influence of interfering species on the response currents of the PAn-PAA37/Au electrode after the addition of 1 mM H_2_O_2_ in PBS at pH 7.0. **(B)** The stability of PAn-PAA37/Au electrode based on the number of days the electrode was stored at 30 °C.

The detection of H_2_O_2_ at negative potentials is essential to avoid the influence of interfering substances, such as ascorbic acid (AA) and uric acid (UA). The potentials required to directly oxidize AA and UA occur at 0.25 V and 0.54 V (Ag/AgCl), respectively [46]. The selectivity of the H_2_O_2_ sensor and its susceptibility to interferences were investigated using the PAn-PAA37/Au electrode. The amperometric response of the sensor to the consecutive injection of 1 mM H_2_O_2_, 1 mM of UA, and 1 mM AA at an applied potential of −0.5 V (Ag/AgCl) is shown Figure 8A. It was evident that the effects of the interfering species were negligible, indicating that the proposed sensor possessed high selectivity.

For enzyme-based sensors, modified electrodes are often stored at 4 °C to retain their activity. The long-term stability of the PAn-PAA37/Au electrode was investigated by storing it at 30 °C in ambient atmosphere for various periods of time. The electrode exhibited very good stability, detectability and repeatable currents. The response current of the sensor in the presence of 1 mM H_2_O_2_ maintained 83.6% of its initial response after 30 days (Figure 8(B)); this result was attributed to the intrinsic thermal stability of PAn.

## 4. Conclusions

An enzyme-free H_2_O_2_ sensor based on a PAn-PAA/Au electrode has been proposed. PAA possesses a carboxyl group on its side chain that could not only acid-dope PAn but also greatly enhance the electroactivity of PAn in a neutral environment. In the presence of H_2_O_2_, the carboxyl group of PAA could react with H_2_O_2_ to form a peroxy acid group. The imines on the main chain of PAn were then oxidized by the peroxy acid group to form an *N*-oxide. Subsequently, the *N*-oxide reverted to its native form via electrochemical reduction, producing a detectable signal. The intrinsic properties of PAn granted the PAn-PAA composite a higher concentration of electroactive imines and a greater stability. The blending of PAA with PAn also produced a composite film with a porous structure, which greatly increased the contact surface area between the PAn-PAA/Au electrode and H_2_O_2;_ this contact enhanced the sensitivity (417.5 μA/mM-cm^2^) and lowered the detection limit (0.02 mM). These results illustrate a potential method for the development of electrode materials that can behave as enzyme-free H_2_O_2_ sensors with favorable sensitivity, selectivity, and stability.

## Figures and Tables

**Table 1.** The characteristics of the H_2_O_2_ sensors based on PAn-PAA/Au electrodes.

Sensor	Detection limit (mM)	Detection range (mM)	Sensitivity (μA/mM-cm^2^)	Response time (s)	R^2^
PAn-PAA28	0.04	0.8–12	553.9	4.98	0.994
PAn-PAA37	0.02	0.04–12	417.5	4.76	0.995
PAn-PAA55	0.06	0.1–10	379.4	3.18	0.995
